# Establishment of Canine Transitional Cell Carcinoma Cell Lines Harboring BRAF V595E Mutation as a Therapeutic Target

**DOI:** 10.3390/ijms22179151

**Published:** 2021-08-25

**Authors:** Hyojik Jung, Kieun Bae, Ja Young Lee, Jung-Hyun Kim, Hyun-Jung Han, Hun-Young Yoon, Kyong-Ah Yoon

**Affiliations:** 1 Department of Veterinary Biochemistry, College of Veterinary Medicine, Konkuk University, Seoul 05029, Korea; jhj2699@konkuk.ac.kr (H.J.); kieun86@konkuk.ac.kr (K.B.); jayoung5573@konkuk.ac.kr (J.Y.L.); 2 Department of Veterinary Internal Medicine, College of Veterinary Medicine, Konkuk University, Seoul 05029, Korea; junghyun@konkuk.ac.kr; 3 Department of Veterinary Emergency and Critical Care, College of Veterinary Medicine, Konkuk University, Seoul 05029, Korea; ab1234@konkuk.ac.kr; 4 Department of Veterinary Surgery, College of Veterinary Medicine, Konkuk University, Seoul 05029, Korea; yoonh@konkuk.ac.kr

**Keywords:** dog, transitional cell carcinoma, canine BRAF V595E, cancer cell line, metastatic lymph node

## Abstract

Transitional cell carcinoma (TCC) is the most common malignant tumor of the canine urinary tract and tends to have a poor prognosis due to its invasive potential. Recent studies have reported that up to 80% of canine urothelial carcinoma has the BRAF V595E mutation, which is homologous to the human V600E mutation. Activating the BRAF mutation is an actionable target for developing effective therapeutic agents inhibiting the BRAF/mitogen-activated protein kinase (MAPK) pathway in canine cancer as well as human cancer. We established novel canine TCC cell lines from two tumor tissues and one metastatic lymph node of canine TCC patients harboring the BRAF V595E mutation. Tumor tissues highly expressed the BRAF mutant and phosphorylated extracellular signal-related kinases (ERK)1/2 proteins. The derived cell lines demonstrated activated MAPK pathways. We also evaluated the cell lines for sensitivity to BRAF inhibitors. Sorafenib, a multiple kinase inhibitor targeting RAF/vascular endothelial growth factor receptor (VEGFR), successfully inhibited the BRAF/MAPK pathway and induced apoptosis. The established canine TCC cell lines responded with greater sensitivity to sorafenib than to vemurafenib, which is known as a specific BRAF inhibitor in human cancer. Our results demonstrated that canine TCC cells showed different responses compared to human cancer with the BRAF V600E mutation. These cell lines would be valuable research materials to develop therapeutic strategies for canine TCC patients.

## 1. Introduction

Transitional cell carcinoma (TCC) is the most common malignant tumor of the canine urinary tract [1,2]. Recently, 80% of canine urothelial carcinoma was reported to have the BRAF V595E mutation, which is homologous to the human V600E mutation [3,4,5]. BRAF is a serine/threonine kinase and its activating mutations lead to constitutive activation of the MAPK pathway, including the ERK1/2 [6,7]. The BRAF V600E mutation is frequently detected in human cancer, especially thyroid carcinoma, melanoma, and non-small cell lung cancer but it is uncommon in urothelial carcinoma [8,9,10,11].

Non-mutated BRAF is activated by the phosphorylation of Thr599 and Ser602 induced by RAS [12]. However, the substitution of valine with glutamic acid at amino acid 600 (V600E) mimics the phosphorylation of Thr599 and Ser602. This mutation activates BRAF constitutively and induces MAPK pathway activation independent of RAS [13]. In canine cancer, which shares similarities with human cancer, activating BRAF mutations play an important role in driver alteration by using the same signaling pathways [14,15].

In human medicine, small molecule inhibitors vemurafenib and dabrafenib targeting the BRAF V600E mutant are approved by the Food and Drug Administration (FDA) [16,17,18]. Previous reports confirmed the effectiveness of kinase inhibitors in human melanoma patients harboring BRAF V600E mutations [19,20]. Although drug resistance has been considered a major obstacle in therapeutic strategies [21,22], RAF inhibitors are still valid therapeutic drugs due to the invention of next-generation kinase inhibitors or combination therapy [23,24]. Likewise, in veterinary medicine, small molecule inhibitors can also be potent anti-cancer drugs for companion animals with spontaneous tumors. Canine TCC is a type of hard-to-treat cancer because of its high invasiveness and metastasis, as well as its associated surgical difficulties. Therefore, it is necessary to develop specific therapeutic agents targeting BRAF mutations for canine TCC patients and TCC cell lines that would be useful research material.

In addition, canine TCC harboring BRAF mutations will be a unique model for human cancers with BRAF signaling activation in the field of comparative oncology. As dogs share life environments with humans, naturally occurring cancers in dogs provide beneficial insight into cancer development and disease progression in humans. Cross-species comparative studies have revealed that canine cancers were relevant to human cancers, demonstrating conserved driver oncogenes and signaling pathways [25,26,27]. Translational cancer research to investigate novel therapeutic agents or drug sensitivity and resistance mechanisms could be done with canine cancer cell lines. 

Although there have been some reports on the establishment of canine TCC cell lines [28,29], the simultaneous establishment of paired cell lines from an original tumor mass and the metastatic region of a patient is rare. In this study, we established not only novel canine TCC cell lines (KU-CTCC-001 and KU-CTCC-002), but also an additional cell line from a metastatic lymph node (KU-CTCC-001-LM3). All cell lines harbor the BRAF V595E mutation. 

The aim of this study was to characterize novel canine TCC cell lines harboring the BRAF V595E mutation. We investigated their morphology, molecular expression, proliferation, and tumorigenicity. We also compared the anti-tumor effects of BRAF inhibitors sorafenib and vemurafenib in the canine TCC cell lines. This report suggests new research materials and therapeutic strategies for canine TCC patients.

## 2. Results

### 2.1. Novel Cell Lines Were Established from Canine TCC Patients 

**Figure 1:**
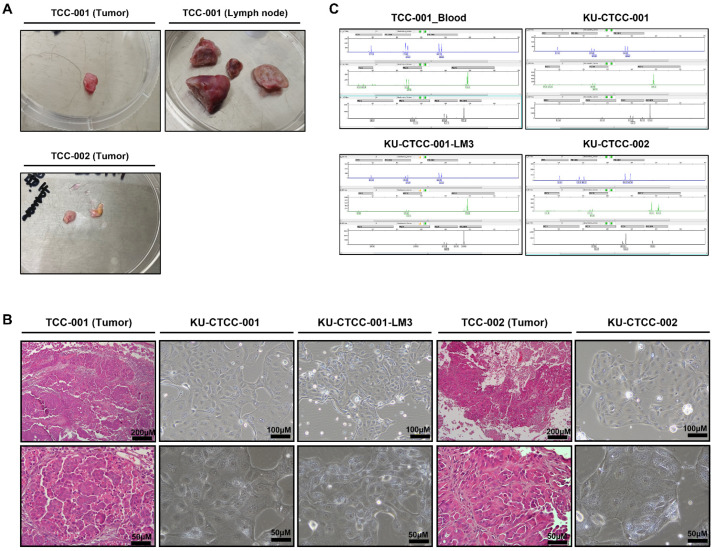
Morphology of original patient tissues and established cell lines. (**A**) Surgically resected tumor tissues collected from canine TCC patients. Tumor tissues were dissociated by mincing and lysed with enzyme. (**B**) Comparison between hematoxylin and eosin (H&E)-stained slides of tumor tissues and cultured cells. All cell lines retained their original morphology. (**C**) Results of STR analysis. The profile showed that KU-CTCC-001 cells and KU-CTCC-001-LM3 cells were derived from the same patient, while KU-CTCC-002 cells were not. They were not contaminated by other sources. TCC-001-Blood represents blood sample derived from patient TCC-001.

A 10-year-old Jindo dog (Patient TCC-001) and an 11-year-old Maltese (Patient TCC-002) were diagnosed with TCC and underwent surgery. The tumor tissue and metastatic sub-lumbar lymph node of patient TCC-001 and tumor tissue of patient TCC-002 were collected after surgery (Table 1, Figure 1A). All tissues were dissociated into single cells. Over 5–6 passages, the tumor cells were discriminated from fibroblasts and became stabilized. The cells were cultured as substrate-adherent cells and grew as a monolayer. The majority of the tumor cells showed a round-to-oval shape but exhibited a spindle shape as they packed together (Figure 1B, Supplementary Figure S1). Similar to the histological characteristics of transitional epithelium, the cytoplasm size varied among the cells. To confirm whether they retained original morphologic features, we compared the cell morphology to hematoxylin and eosin (H&E)-stained original tumor tissue. Each cell line retained the morphology of the original tissue from which it was derived. In addition, KU-CTCC-001-LM3, the cell line from the metastatic lymph node showed a morphology similar to that of the cell line from the original tumor mass. The KU-CTCC-002 looked similar to KU-CTCC-001 but they were slightly larger. We performed a short tandem repeat (STR) analysis to authenticate each cell line (Figure 1C). Canine-specific loci were distributed in a heterozygous pattern between the two patients, without cross-contamination. The STR profile confirmed that KU-CTCC-001 and KU-CTCC-001-LM3 were derived from the same patient and KU-CTCC-002 had a different origin.

### 2.2. All Cell Lines Have BRAF V595E Mutations and MAPK Pathway Activation 

**Figure 2:**
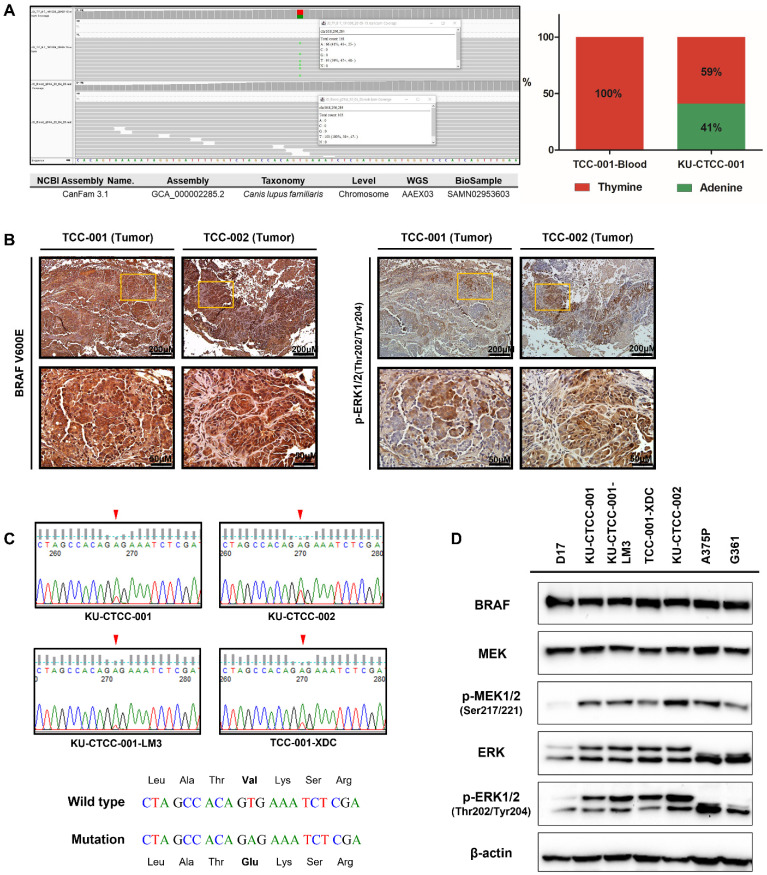
Detection of BRAF V595E mutations and MAPK pathway activation. (**A**) Somatic variant-calling results of the *BRAF* gene in KU-CTCC-001. In contrast to the blood results, KU-CTCC-001 cells showed heterozygous mutations. Wild-type (thymine) reads were mapped to the reference sequence more than mutant (adenine) reads. The ratio of wild-type to mutants was 59:41. (**B**) Immunohistochemical (IHC) staining of BRAF mutations and phosphorylated ERK1/2 protein. Tumor tissues were strongly stained with anti-BRAF V600E antibody, suggesting the presence of V595E mutations. Phosphorylated ERK1/2 protein was also highly expressed in the tumor tissues. (**C**) Detection of BRAF V595E mutations by Sanger sequencing. Heterozygous mutations were confirmed not only in KU-CTCC-001 cell lines but also in the other cell lines. Furthermore, tumor-dissociated cells derived from the xenograft mouse model injected with KU-CTCC-001 cells (TCC-001-XDC) showed a similar ratio of wild-type to mutants as the original cell line (KU-CTCC-001). (**D**) MAPK pathway activation at the protein level. The expression of phosphorylated MEK1/2 protein and phosphorylated ERK1/2 protein was similar to that of human BRAF V600E mutant cell lines (A375P and G361) and higher than that of the D17 cell line.

To screen for cancer driver genes, we performed whole-exome sequencing (WES) of KU-CTCC-001 (Supplementary Figure S2, Supplementary Table S1). Of the total 10,107 somatic variants, we selected 3980 protein-coding variants and excluded 2314 synonymous mutations. Based on the Cancer Gene Census (COSMIC) database, we extracted the genes correlated with cancer (319 mutations of 71 genes). Then, we selected the tumor suppressor genes and oncogenes involved in the MAPK pathway (based on the Kyoto Encyclopedia of Genes and Genomes (KEGG)). Filtering by allele fractions (>0.05) and PROVEAN scores (<−2.500), we finally selected significant genes and mutations. In the profiles, we found the BRAF V595E mutation, which is already known as a driver gene in canine TCC, to be the most significant mutation. The mutant peak (c.1784T>A) was heterozygous and the ratio of wild-type to mutants was 59:41. Of the total 161 reads, 95 wild-type (thymine) reads and 66 mutant (adenine) reads were mapped to the reference sequence (Figure 2A). Immunohistochemical (IHC) staining with anti-BRAF V600E antibody displayed strong positive staining in the tumor tissues. The amino acid sequence of BRAF protein is 99% identical between humans and dogs; in particular, amino acid sequences encoded by exon 15 are identical. Therefore, positive staining with anti-BRAF V600E antibody suggests the presence of a canine V595E mutation (Figure 2B). The substitution of codon 595 in the BRAF gene (GTG > GAG) was confirmed in all TCC cell lines using Sanger sequencing (Figure 2C). Furthermore, the same mutation was also detected in tumor-dissociated cells derived from the xenograft mouse model injected with KU-CTCC-001 cells (called TCC-001-XDC). Expression of BRAF, ERK and MEK was examined in mRNA level and protein level (Figure 2D, Supplementary Figure S3). Activation of the MAPK signaling pathway in the TCC cell lines was suggested by a high level of phosphorylated MEK1/2 (p-MEK1/2) protein and phosphorylated ERK1/2 (p-ERK1/2) protein (Figure 2D). They were expressed to levels similar to those of human melanoma cell lines harboring the BRAF V600E mutant (A375P and G361) and higher than that of D17 cells, a canine osteosarcoma cell line that was used as a negative control. The high expression of phosphorylated ERK1/2 protein was also displayed in tumor tissues by IHC analysis (Figure 2B). 

### 2.3. In Vitro Evaluation of General Growth Characteristics 

**Figure 3:**
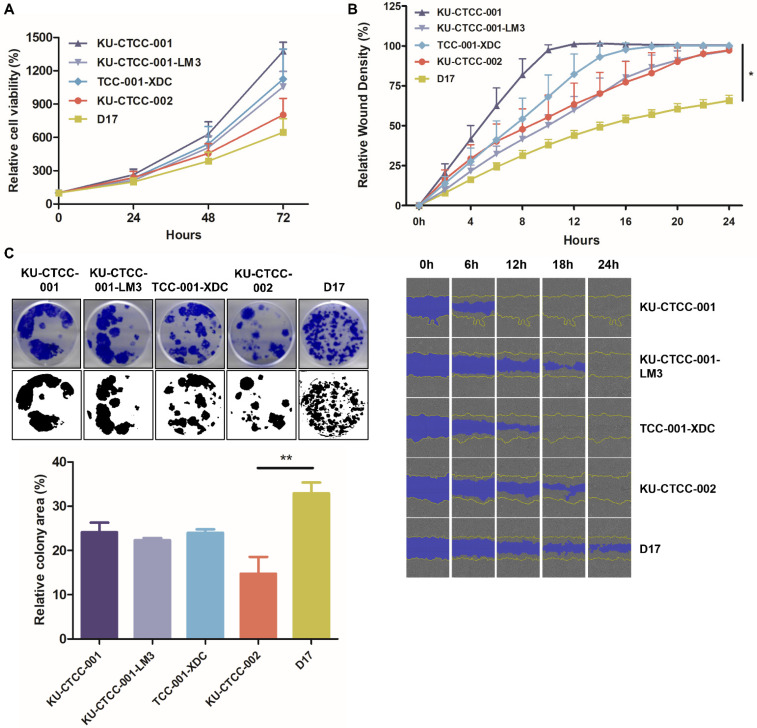
General growth characteristics of TCC cell lines. (**A**) Comparison of proliferation ability in cell lines using the MTT assay. All canine TCC cell lines proliferated faster than D17 cells (*p* = 0.2441, two-way repeated measure ANOVA). KU-CTCC-001 proliferated the fastest. There was no significant difference between KU-CTCC-001 and TCC-001-XDC. Error bars represent SEM. (**B**) The results of the wound-healing assay. All TCC cell lines filled the wound area within 24 h. Error bars represent SD. Statistical significance was determined by one-way ANOVA; * *p* < 0.05 (**C**) Representative images of the colony-forming assay. Error bars represent SEM. Statistical significance was determined by one-way ANOVA; ** *p* < 0.01. All experiments were performed in triplicate.

All TCC cell lines proliferated faster than D17 cells, which were used as a comparison group (Figure 3A). KU-CTCC-001 proliferated the fastest among them. There was no significant difference in the proliferation rate between KU-CTCC-001 and TCC-001-XDC. The doubling times for cell lines were as follows; 17.7 ± 1.2 h (h) for KU-CTCC-001, 19.1 ± 1.2 h for KU-CTCC-001-LM3, 19.7 ± 2.9 h for TCC-001-XDC, and 20.0 ± 1.5 h for KU-CTCC-002. As the metastatic potential is a characteristic of TCC, we examined migratory ability by the wound-healing assay and evaluated the expression of epithelial-mesenchymal transition (EMT) markers in the TCC cell lines (Figure 3B, Supplementary Figures S1 and S4). All TCC cell lines covered the wound area within 24 h (h) and KU-CTCC-001 was the fastest. However, CDH1 expression was higher in the TCC cell lines than in D17 or human BRAF V600E mutant cell lines (A375P and G361) and the expression of other mesenchymal markers (CDH2, vimentin, and Slug) was lower than that of the control cells except for Snail. Given these results, TCC cell lines have high proliferative activity and migration properties, and the EMT mechanism did not play an important role in our cell lines. We also evaluated colony-forming ability in the cell lines. Although the TCC cell lines exhibited lower colony-forming ability than the D17 cells, KU-CTCC-001 showed higher colony-forming ability than KU-CTCC-002 (Figure 3C). There was no significant difference between KU-CTCC-001 and TCC-001-XDC.

### 2.4. Tumorigenicity Is Confirmed in an In Vivo Xenograft Mouse Model

**Figure 4:**
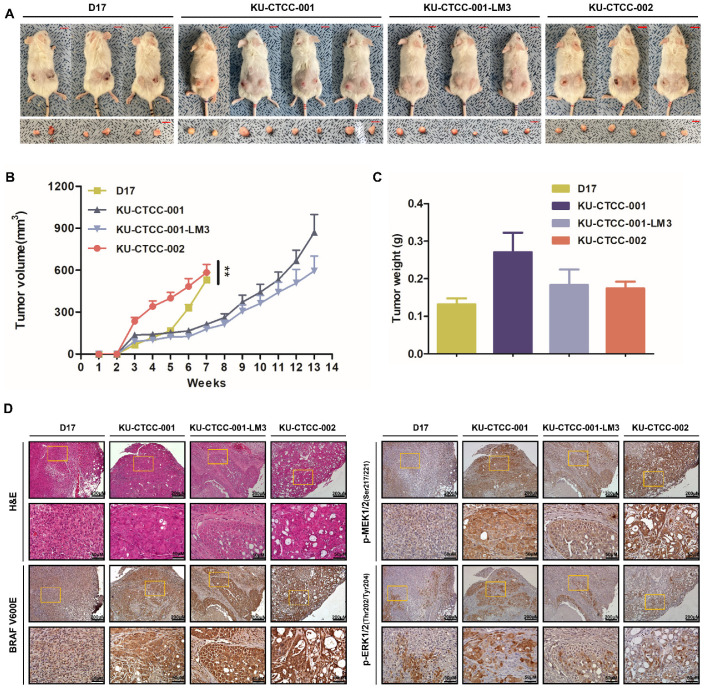
Tumorigenicity in the in vivo xenograft mouse model. (**A**) NOG xenograft mouse model. All cell lines showed tumorigenicity. Scale bar: 1 cm. (**B**) Growth curve of subcutaneous tumors after injection. Tumors in mice injected with KU-CTCC-002 grew faster than those of the other cell lines. As the tumor surface scar became severe, we euthanized xenograft mice injected with KU-CTCC-002 and D17 cells seven weeks after injection, before the tumor volume approached 1500 mm^3^. Error bars represent SEM. Statistical significance was determined by two-way repeated measure ANOVA; ** *p* < 0.01. (**C**) Tumor weight after necropsy. Error bars represent SEM. (**D**) Representative H&E and immunohistochemistry images at low (×100, scale bar = 200 μm) and high magnification (×400, scale bar = 50 μm). The tumors injected with TCC cell lines were more positively stained by anti-BRAF V600E antibody than those of the D17 cells. Positive staining of tumors with p-MEK1/2 and p-ERK1/2 antibodies indicated MAPK pathway activation.

In vivo tumorigenicity was investigated using a xenograft mouse model. We injected the cell lines subcutaneously into athymic nude (NU(NCr)-Foxn1nu) and NOG (NOD.Cg-Prkdcscid Il2rgtm1Sug/Jic) mice. All cell lines demonstrated tumorigenicity in both strains (Figure 4A; Supplementary Figure S5A). Significant body weight loss was not observed in either strain (data not shown). In contrast to NOG, in which all inoculated sites showed 100% tumorigenicity (D17 (6/6 inoculated sites), KU-CTCC-001 (8/8), KU-CTCC-001-LM3 (6/6), and KU-CTCC-002 (6/6)), the athymic nude mice showed different rates according to the cell lines (D17 (6/8), KU-CTCC-001 (8/8), KU-CTCC-001-LM3 (2/10), and KU-CTCC-002 (6/6)). In both strains, the tumors grew the fastest in the mice injected with the KU-CTCC-002 cells compared to the other cell lines (Figure 4B; Supplementary Figure S5A). Although slower than the tumors in mice injected with D17 cells, tumor growth in the mice injected with KU-CTCC-001 was faster than in those injected with KU-CTCC-001-LM3. Originally, necropsies were done before the tumor volume reached 1500 mm^3^ but as the tumor surface scar became severe, we euthanized the NOG mice injected with KU-CTCC-002 and D17 cells seven weeks after the injections. There was no metastasis in either mouse strain. Tumor weight and volume were proportional (Figure 4C). To investigate the histological features, subcutaneous tumor sections were examined by H&E staining (Figure 4D, Supplementary Figure S5B). In contrast to the tumors in mice injected with D17 cells, which were tightly packed with tumor cells, the tumor cells were surrounded by connective tissues in those injected with TCC cell lines. All TCC cell line tumors had eosinophilic amorphous necrosis in the center and the majority of the live cells were found in the margin of the tumors. Tumors that were formed by KU-CTCC-002 cells had especially severe necrosis. The necrotic cells exhibited a loss of nuclei and increased eosinophilia and fragmentation. MAPK pathway activation was evaluated by IHC analysis using anti-BRAF V600E, p-MEK1/2, and p-ERK1/2 antibodies. The tumors injected with the TCC cell lines were more positively stained by anti-BRAF V600E antibody and p-MEK1/2 antibody than the negative controls (the tumors injected with D17 cells). In addition, the tumors expressed a high level of phosphorylated ERK1/2 protein, which were stained positive with the p-ERK1/2 antibody. The findings suggest that the tumors derived from the xenografts also exhibited MAPK pathway activation.

### 2.5. Canine TCC Cell Lines Are More Sensitive to Sorafenib than Vemurafenib 

**Figure 5:**
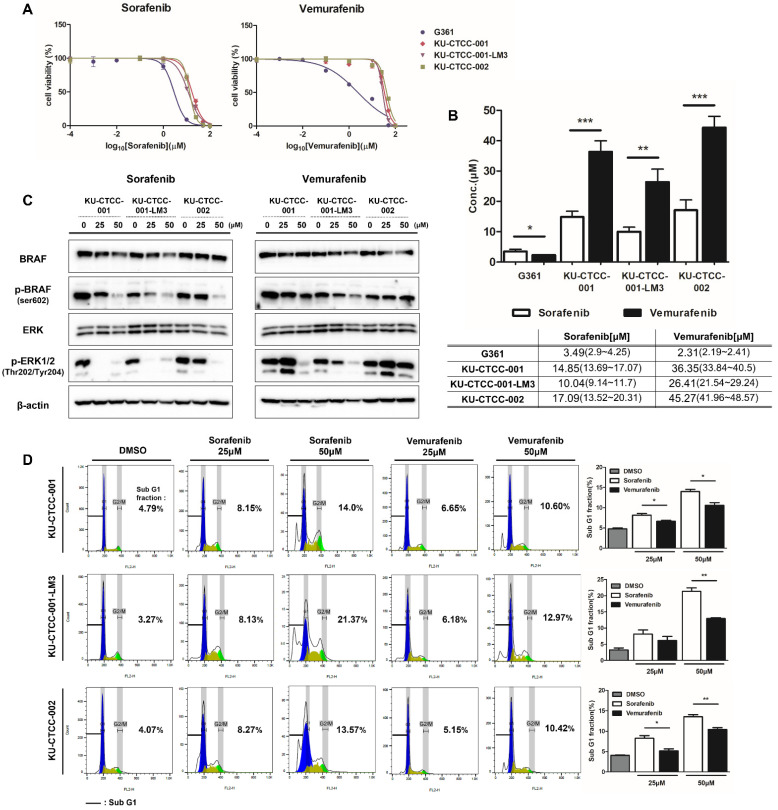
Drug sensitivity to BRAF inhibitors. (**A**) Drug response curve to sorafenib and vemurafenib. In contrast to the human cell line G361, sorafenib induced cell death at lower concentrations than vemurafenib in canine TCC cell lines. (**B**) IC50 values in the cell lines. The IC50 values of sorafenib were lower than those of vemurafenib in canine TCC cell lines. For both drugs, the IC50 value of KU-CTCC-002 was the highest, and that of KU-CTCC-001-LM3 was the lowest. Error bars represent SEM. Statistical significance was determined by unpaired Student’s *t*-test; * *p* < 0.05, ** *p* < 0.01, *** *p* < 0.001. (**C**) Comparison of MAPK pathway inhibition between both drugs at the protein level. Sorafenib inhibited phosphorylated BRAF and phosphorylated ERK1/2 protein more effectively than vemurafenib at the same concentration. (**D**) Evaluation of apoptosis through cell cycle analysis. The degree of apoptosis caused by the drugs was evaluated by calculating the sub-G1 fraction. In the graph, the percentage number represents the sub-G1 fraction. Sorafenib induced more apoptosis than vemurafenib. Error bars represent SEM (*n* = 3). Statistical significance; * *p* < 0.05, ** *p* < 0.01. All experiments were performed in triplicate.

We examined the effect of sorafenib (BAY 43-9006), a multiple kinase inhibitor targeting RAF/VEGFR, on canine TCC cell lines. We also compared the sensitivity of sorafenib with vemurafenib (PLX4032) since vemurafenib is a specific inhibitor of BRAF mutations. G361, a human melanoma cell line harboring heterozygous BRAF V600E mutations, was used as a control. Cell viability was measured after treatment with drugs at each concentration and the half maximal inhibitory concentration (IC50) values were compared between drugs (Figure 5A,B). As expected, the G361 cells responded to both inhibitors and had a lower IC50 for vemurafenib than for sorafenib. In contrast to G361, the canine TCC cell lines were more sensitive to sorafenib than vemurafenib. KU-CTCC-002 had higher IC50 values for sorafenib and vemurafenib than those of the other cell lines. KU-CTCC-001-LM3 showed the lowest IC50s for both drugs. To examine the inhibitory effect of each drug on protein levels, we compared the expression level of phosphorylated BRAF (p-BRAF) and phosphorylated ERK1/2 proteins by Western blot analysis after drug treatment (Figure 5C). Sorafenib inhibited the MAPK pathway more effectively than vemurafenib. The degree of apoptosis was also compared by calculating the sub-G1 fraction of the cell cycle (Figure 5D). Our results showed that sorafenib induced apoptosis more than vemurafenib in canine TCC cells and the gap was more obvious at higher concentrations. 

## 3. Discussion

Transitional cell carcinoma, also called urothelial carcinoma, is the most common malignancy of the canine urinary tract, affecting tens of thousands of dogs worldwide every year [30]. Most bladder cancer is intermediate to high-grade invasive TCC (>90% of the cases) [31]. The majority of the tumors are located in the trigone region of the bladder. However, due to its invasive characteristics, more than half of TCCs have been reported to involve the urethral region (56%) [31]. Moreover, metastasis commonly occurs in various regions such as regional lymph nodes, lungs, or skin [30].

In our cases, the majority of the original tumor of patient TCC-001 was located in the urethra. However, as the tumor grew invasively, it extended to the trigone area. The tumors of patient TCC-002 were distributed widely from the bladder trigonal region to the proximal urethra including the prostate. In addition, patient TCC-002 showed severe hydronephrosis due to ureteral dilation. The trigonal mass obstructed the ureterovesical junction and induced ureteral dilation. Both patients had regional lymph node metastasis, but the metastatic lymph nodes of patient TCC-002 were not available for further experiments.

There are diverse treatment methods for TCC including surgery, radiation therapy, chemotherapy, and a combination of these. In contrast to human bladder cancer, which requires complete cystectomy as a front-line treatment, surgical excision is not recommended as a front-line treatment for canine TCC. Due to the trigonal location of the tumors and the frequency of urethral involvement, complete surgical excision is difficult [31,32]. Except for inevitable conditions such as urethral stricture inducing severe dysuria, surgical excision is not recommended. Therefore, chemotherapy using single agent or various combination therapies have been tried for TCC patients [30,33]. However, significant increases in survival time have not been observed and toxicity has been considered a limitation. This suggests that additional therapeutic strategies for canine TCC are needed [34]. 

The canine BRAF V595E mutation, which is homologous to the human BRAF V600E mutation, is known as a common driver gene mutation in canine TCC [3,4,5]. Given that the oncogenic effects of the BRAF mutation are the same as those in human cancer, inhibitors targeting activation of the BRAF/MAPK pathway could also be effective for treating dog cancer as well as human cancer. In human medicine, vemurafenib is a specific BRAF V600E mutant target inhibitor [16,17,18]. Previous studies demonstrated various sensitivities of vemurafenib to canine TCC harboring BRAF V595E mutations [3,15]. Because sorafenib was known as the first RAF kinase inhibitor [8,35], we compared the sensitivity between the two drugs. Our TCC cell lines were more sensitive to sorafenib than vemurafenib. This result suggests various reasons including the following: (1) other genetic influences on the oncogenic effects in dogs, (2) an innate resistance mechanism to vemurafenib, and (3) differences in drug binding affinity to the BRAF protein between humans and dogs. To identify the specific mechanisms, additional TCC cell lines and studies are needed. In addition, resistance to BRAF inhibitors has been considered a serious obstacle in human medicine [21,22] and many studies are in progress to overcome resistance [23,24,36]. From this perspective, our TCC cell lines harboring the BRAF V595E mutation would be a novel tool for researching drug mechanisms and resistance.

Several previous studies have reported the establishment of canine TCC cell lines [28,29]. However, reports of the establishment of paired cell lines from the original tumor mass and the metastatic region of a patient are rare. In addition, canine TCC cell lines published in such papers also had BRAF V595E mutations, but no reports simultaneously showed drug responses targeting such mutations. Here, in this study, we established three canine TCC cell lines including two cell lines derived from tumor tissues and a cell line from a metastatic lymph node. All TCC cell lines showed heterozygous BRAF V595E mutations and MAPK pathway activation.

KU-CTCC-001 and KU-CTCC-002, derived from the original tumors of patients, showed different growth characteristics. KU-CTCC-001 proliferated faster than KU-CTCC-002 in vitro. However, it showed the opposite characteristics in vivo. The tumors that were formed by KU-CTCC-002 cells had a large area of necrosis, numerous immune cells, and connective tissues in the xenograft tumor, suggesting that cancer cells significantly interacted with other cells such as fibroblasts or macrophages in vivo. In addition, as we established paired cell lines from the original tumor mass and a metastatic region, we compared them in almost all experiments we performed. KU-CTCC-001 proliferated faster than its metastatic cell line KU-CTCC-001-LM3 both in vitro and in vivo. Even though we concluded that KU-CTCC-001-LM3 did not have more oncogenic characteristics than KU-CTCC-001 based on these results, further investigation should be conducted on these relationships.

Moreover, to confirm whether the oncogenic characteristics of the cells changed after tumorigenesis in vivo, we dissociated the tumors derived from xenograft mice injected with KU-CTCC-001 to single cells (TCC-001-XDC) and compared the two cell lines. There was no significant difference between the two cell lines in the ratio of wild-type to mutant, MAPK pathway activation at the protein level, and in vitro evaluation of growth characteristics. There was also no metastasis of any xenografts formed by injection of the TCC cell lines. Although xenografts from KU-CTCC-001 and KU-CTCC-001-LM3 injections showed no metastatic regions until the tumor volume reached 1500 mm^3^, mice injected with KU-CTCC-002 cells were euthanized before the tumor volume reached 1500 mm^3^, so longer monitoring may be required to detect metastasis.

In terms of comparative oncology, these TCC cell lines would be also valuable research materials. Canine invasive urethral TCC mimics that of humans in pathology, local invasion, frequency of distant metastasis, and response to chemotherapy [37]. Currently, human patients with invasive urethral carcinoma in the United States face various challenges including a 50% fatality rate, above 16,000 deaths per year, reduced quality of life, and the financial burden of treatment costs [37]. Thus, canine TCC models are useful and valuable in investigating new treatment strategies. Dhawan et al. suggested that the DNA methyltransferase 1 (DNMT1) gene, which is overexpressed in both human and canine TCC, was an effective drug target in both species, demonstrating antiproliferative effects by the demethylating agent 5-azacitidine [38].

In conclusion, we established novel canine TCC cell lines from two tumor tissues and one metastatic lymph node of canine TCC patients harboring BRAF V595E mutations. They all exhibited MAPK pathway activation and tumorigenicity in in vivo xenografts. In addition, we evaluated our cell lines for sensitivity to two RAF inhibitors, sorafenib and vemurafenib. In contrast to the human cell line, sorafenib showed more anti-tumor effects than vemurafenib. These cell lines would be valuable research materials for investigating therapeutic agents or studying drug mechanisms in both humans and dogs. In addition, this study suggests a new therapeutic strategy for canine TCC patients.

## 4. Materials and Methods

### 4.1. Patient Information and Primary Cell Culture

A 10-y-old spayed female Jindo dog (Patient TCC-001) and an 11-y-old neutered male Maltese (Patient TCC-002) came to Konkuk University Veterinary Medical Teaching Hospital (KU-VMTH, Seoul, Korea) and were pathologically diagnosed with TCC [39]. Patient TCC-001 had metastasis at the sub-lumbar lymph nodes. Surgically resected specimens were collected with the consent of the owners under the approval of the Ethical Committee of Konkuk University, Seoul (KU18141, KU19189). After surgery, each tumor sample was harvested and lysed by collagenase II (Life Technologies, Carlsbad, CA, USA), hyaluronidase, and Ly27632 (Sigma-Aldrich, St. Louis, MO, USA) for 30 min at 37 °C [40]. After filtering through a 70-μm cell strainer (BD Biosciences, San Diego, CA, USA), the tumor samples were dissociated into single cells and cultured in Advanced Dulbecco’s modified Eagle’s medium (DMEM)/F12 supplemented with 10% fetal bovine serum (FBS), 10 mM HEPES, Glutamax (Thermo Fisher Scientific, Waltham, MA, USA), and Zell shield (Minerva Biolabs, Berlin, Germany). D17, the canine osteosarcoma cell line, was purchased from the American Type Culture Collection (Manassas, VA, USA) and cultured in Eagle’s Minimum Essential Medium with 10% heat-inactivated FBS and 1% antibiotics. Human cell lines (A375P and G361) were purchased from the Korean Cell Line Bank. They were cultured in DMEM (A375P) or RPMI1640 (G361) containing 10% heat-inactivated FBS and 1% antibiotics. All cells were cultured at 37 °C in a humidified atmosphere of 5% CO_2_. The growth rate of each cell line was evaluated by counting cell numbers at 24-h intervals for 5 days. Cell doubling time was calculated from the growth rate.

### 4.2. Sequencing and STR Analysis

BRAF V595E mutations were detected by Sanger sequencing and whole-exome sequencing (WES). Genomic DNA was extracted from the cells using a QIAamp DNA Mini Kit (Qiagen, Valencia, CA, USA) and BRAF exon 15, in which the V595E mutation is located, was amplified using BRAF primers (forward, 5ʹ-ATTTCAAGCCCCCAAAATCT-3ʹ; reverse, 5ʹ-GTAGCACCTCAGGGTCCAAA-3ʹ). The WES details are described in the Supplementary Materials and Methods.

StockMarks^®^ Kits for Dogs (Applied Biosystems, Foster City, CA, USA) were used to analyze the STRs of genomic DNA and performed according to the manufacturer’s protocols.

### 4.3. Colony-Forming Assay and Migration Assay (Wound-Healing Assay)

To evaluate colony-forming ability, 7 × 10^2^ cells were seeded in 6-well plates. The medium was changed twice a week. After two weeks, the medium was removed and stained with 0.5% crystal violet solution. The colony-covering area was measured using ImageJ software (National Institutes of Health, Bethesda, MD, USA). We also conducted migration assays to evaluate migratory ability. Cells (2 × 10^4^) were seeded in 96-well plates and incubated for 24 h to form a monolayer of cells. Wounds were made on the cells using a 96-pin Wound Maker and cell migration was monitored using the IncuCyte™ Live-Cell Imaging System (Essen BioScience, Michigan, MI, USA). The images were collected every 2 h and analyzed by the IncuCyte software system. All experiments were performed in triplicate.

### 4.4. Western Blot Analysis

Proteins were extracted in cell lysis buffer containing phosphatase inhibitor cocktail, protease inhibitor, dithiothreitol, and phenylmethylsulfonyl fluoride. All samples were loaded on sodium dodecyl sulfate-polyacrylamide gels in equal amounts, electrophoresed, and transferred to membranes. After blocking with 5% skim milk-Tris-buffered saline with 0.1% Tween 20 detergent for 1h, the membranes were incubated with primary antibodies. Antibodies were purchased from Cell Signaling Technology (Danvers, MA, USA) (MEK1/2 (#4694), p-MEK1/2 (Ser217/221) (#9154), ERK1/2 (#4696), p-ERK1/2 (Thr202/Tyr204) (#4370)), Santa Cruz Biotechnology (Dallas, TX, USA) (BRAF(#sc-55522)); Thermo Fisher Scientific (p-BRAF (ser602) (#PA5-38412)); and Abcam (Cambridge, UK) (BRAF V600E (clone RM8, ab200535)). For detailed information on the EMT antibodies, see Supplementary Materials and Methods.

### 4.5. In Vivo Mouse Xenograft

Cells (5 × 10^6^) were inoculated subcutaneously into the flanks of 6-week-old athymic nude (NU(NCr)-Foxn1nu) and NOG (NOD.Cg-Prkdcscid Il2rgtm1Sug/Jic) mice. We inoculated cells into both sides of the flanks on each mouse. Tumor volume (mm^3^) was measured twice a day and estimated using the equation (A × B^2^)/2 (A, the longest diameter of the tumor and B, the shortest diameter of the tumor). Before exceeding a tumor volume of 1500 mm^3^, the mice were euthanized and necropsies were performed. Tumor tissues in which the cells were subcutaneously inoculated were harvested and dissociated into cells. All animal experiments in this study were performed under the guidelines of the Institutional Animal Care and Use Committee (IACUC) (approval No.: KU21110). 

### 4.6. Hematoxylin and Eosin Staining and Immunohistochemistry 

The samples were fixed in 4% paraformaldehyde and embedded in paraffin. The formalin-fixed paraffin-embedded tissues were sectioned by 4 μm and stained with hematoxylin and eosin. 

For immunohistochemical analysis, antigen retrieval was performed by the microwave method with 0.1 M sodium citrate buffer (pH 6.0). After treating with 3% hydrogen peroxide to inhibit peroxidase activity, the slides were blocked in 1% bovine serum albumin and then incubated with primary antibody overnight. After incubation with a biotinylated secondary antibody, the 3,3′-diaminobenzidine substrate was applied for enzymatic detection. 

### 4.7. MTT Assay and IC50

Cells (1 × 10^4^) were seeded in 96-well plates and incubated overnight at 37 °C in a humidified atmosphere of 5% CO_2_. The medium was replaced with that containing gradient doses of drugs. After 48 h, cell viability was examined using the CellTiter-Glo^®^ Luminescent Cell Viability Assay (Promega, Madison, WI, USA). IC50 values were measured using GraphPad Prism 5 (GraphPad Software Inc., San Diego, CA, USA) with nonlinear regression analysis. Sorafenib (BAY 43-9006) and vemurafenib (PLX4032) were purchased from Selleckchem (Houston, TX, USA). Each drug was serially diluted with dimethyl sulfoxide to the proper concentrations. All experiments were performed in triplicate.

### 4.8. Cell Cycle Assay

The cells were treated with sorafenib and vemurafenib for 48 h. After harvesting, the cells were fixed with 70% ethanol at 4 °C overnight and stained with propidium iodide solution containing RNase A for 30 min. the cells were analyzed by flow cytometry (FACScalibur II, BD Biosciences, MD, USA) and the proportion of cells in the sub-G1 phase was calculated using FlowJo software (Tree Star Inc., Ashland, OR, USA).

### 4.9. Statistical Analysis

All data were statistically analyzed using GraphPad Prism 5 (GraphPad Software Inc., San Diego, CA, USA). The cell growth rates and tumor volume growth in the xenograft model (Figure 3A and Figure 4B) were analyzed by a two-way repeated measure analysis of variance (ANOVA). Other data were analyzed by one-way ANOVA with Tukey’s post-hoc test or unpaired Student’s *t*-test. Statistical significance was indicated at * *p* < 0.05, ** *p* < 0.01, and *** *p* < 0.001.

## Figures and Tables

**Table 1:** Patient information.

Patient ID	Body Weight	Sex ^1^	Age	Breed	Tumor Location	Histologic Diagnosis	Cell Lines
TCC-001	22.8 kg (BCS 5/9)	SF	10y	Jindo dog	Urethra (extending to trigone region of bladder)/Sublumbar lymph nodes	Urothelial Transitional cell carcinoma, non-papillary/infiltrative subtype	KU-CTCC-001/KU-CTCC-001-LM3
TCC-002	3.2 kg (BCS 4/9)	NM	11y	Maltese	Urinary bladder (trigone region) extending to urethra including prostate	Urothelial Transitional cell carcinoma	KU-CTCC-002

^1^ SF = Spayed female, NM = Neutered male.

## Supplementary Materials

Supplementary materials can be found at https://www.mdpi.com/article/10.3390/ijms22179151/s1.
