# Supplementary material for: Establishment of Canine Transitional Cell Carcinoma Cell Lines Harboring BRAF V595E Mutation as a Therapeutic Target
Source: Int J Mol Sci. 2021 Aug 25;22(17):9151. doi: 10.3390/ijms22179151 (PMC8430554; doi:10.3390/ijms22179151)
Supplement: Supplementary file 1 [file ijms-22-09151-s001.zip › ijms-1331561-supplementary.pdf]

Supplementary data

Table of contents

- Supplementary Materials and Methods……………………….2-3
- Supplementary Figure 1……………………………………………4
- Supplementary Figure 2……………………………………………5
- Supplementary Figure 3……………………………………………6
- Supplementary Figure 4……………………………………………7
- Supplementary Figure 5……………………………………………8
- Supplementary Table 1……………………………………………9

Supplementary Materials and Methods

1. Whole-exome sequencing

The quality and quantity of purified DNA were assessed by fluorometry and gel electrophoresis, respectively. Briefly, 500 ng of genomic DNA from each sample was fragmented by acoustic shearing on a Covaris S2 instrument. Fragments of 150 – 200 bp were ligated to Illumina adapters and PCR-amplified. The samples were concentrated to 750 ng in 3.4 μl of distilled water (DW) using a Speedvac (Thermo Scientific) and hybridized with RNA probes using the SureSelect XT Canine All Exon V2 Kit Capture library for 16 – 24 h at 65 °C.

After hybridization, the captured targets were pulled down by biotinylated probe/target hybrids using streptavidin-coated magnetic beads (Dynabeads My One Streptavidine T1; Life Technologies, Ltd.) and buffers. The selected regions were then PCR-amplified using Illumina PCR primers. Libraries were identified with an Agilent TapeStation 4200 using High Sensitivity D 1000 ScreenTape (Agilent, Santa Clara, CA, USA) and the KAPA Library Quantification Kit (Kapa Biosystems, Wilmington, MA, USA).

The high-quality libraries were pooled and sequenced on the Illumina NovaSeq6000 platform (Illumina) with 150 bp paired-ends by following the manufacturer's protocols. Image analysis was performed using NovaSeq6000 control software version 1.3.1 and the output base-calling data were de-multiplexed with bcl2fastq version v2.20.0.422, generating fastQC files.

After somatic variant-calling was performed, we selected genes with protein-coding variants inducing amino acid changes as driver genes causally implicated in cancer based on genes listed in the Cancer Gene Census (COSMIC) database (version 92). Then, the oncogenes involved in the MAPK pathway based on the KEGG pathway and tumor suppressor genes were extracted. Finally, genes and mutations were selected above 0.05 for allele fractions and below -2.500 for PROVEAN scores.

2. Primary cell culture

To investigate EMT marker protein expression, we used the KU-CTCC-001-LM4 cell line, another metastatic cell line, which was also derived from sub-lumbar lymph nodes, as well as KU-CTCC-001-LM3.

3. Primary antibodies for EMT markers

Primary antibodies were purchased from Cell Signaling Technology (CDH1 (#3195), Snail (#3895), Slug (#9585), vimentin (#5741)), Abcam (EpCAM (ab71916)) and Santa Cruz Biotechnology (CDH2 (#sc-393933)).

4. *RT-PCR*

To confirm the mRNA expression level of MAPK pathway, RT-PCR analysis was performed. Primers are designed based on conserved sequence between human and dog. The sequence difference is within 1 base pair. Primer sequences are presented as below table.

Gene	Forward primer (5'-3')	Reverse primer (5'-3')
BRAF	TCAACCACAGGTTTGTCTGC	GATGACTTCTGGTGCCATCC
MEK1	AGTGCAACTCCCCGTACATC	GGCGACATGTAGGACCTTGT
ERK2	TGATCACACAGGGTTCCTGA	TGTGATGGGGATCCAAGAAT
GAPDH	AAGGTCATCCCTGAGCTGAA	GACCACCTGGTCCTCAGTGT

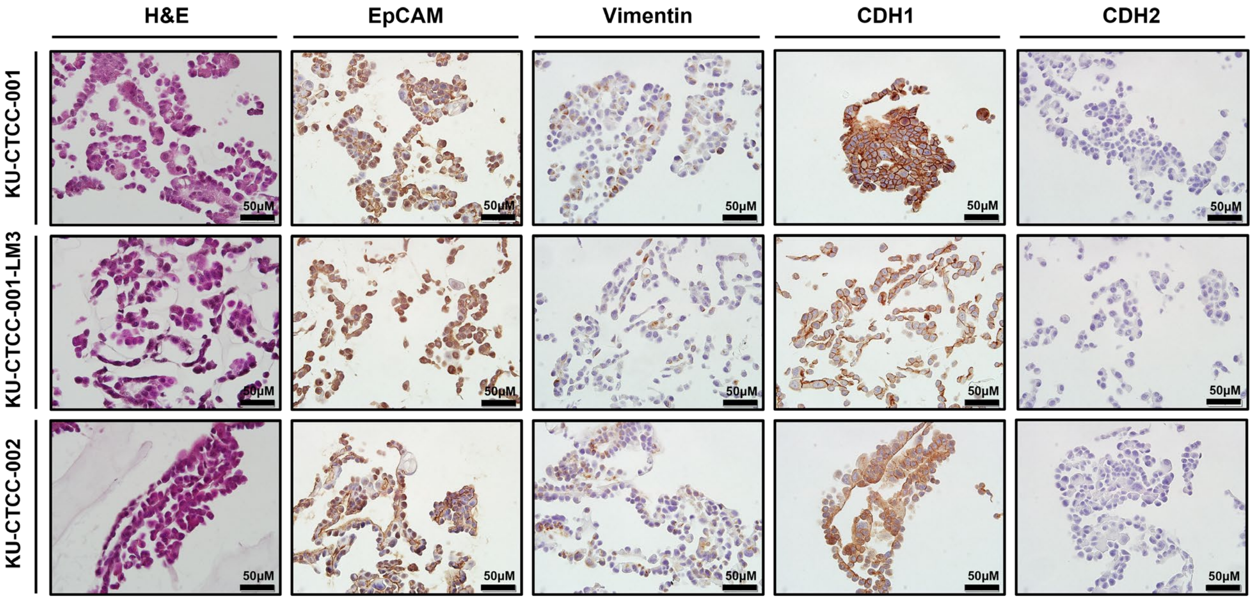

Supplementary Figure 1. H&E and IHC staining images of formalin-fixed paraffin-embedded cell pellet block. Tumor cells are round-to-oval shape and the cytoplasm size varies among cells. High expression of epithelial cell adhesion molecule (EpCAM) and CDH1 represents the epithelial characteristics of the established cell lines. Low immunoreactivity of mesenchymal markers such as vimentin and CDH2 suggests TCC cell lines exhibit low EMT features.

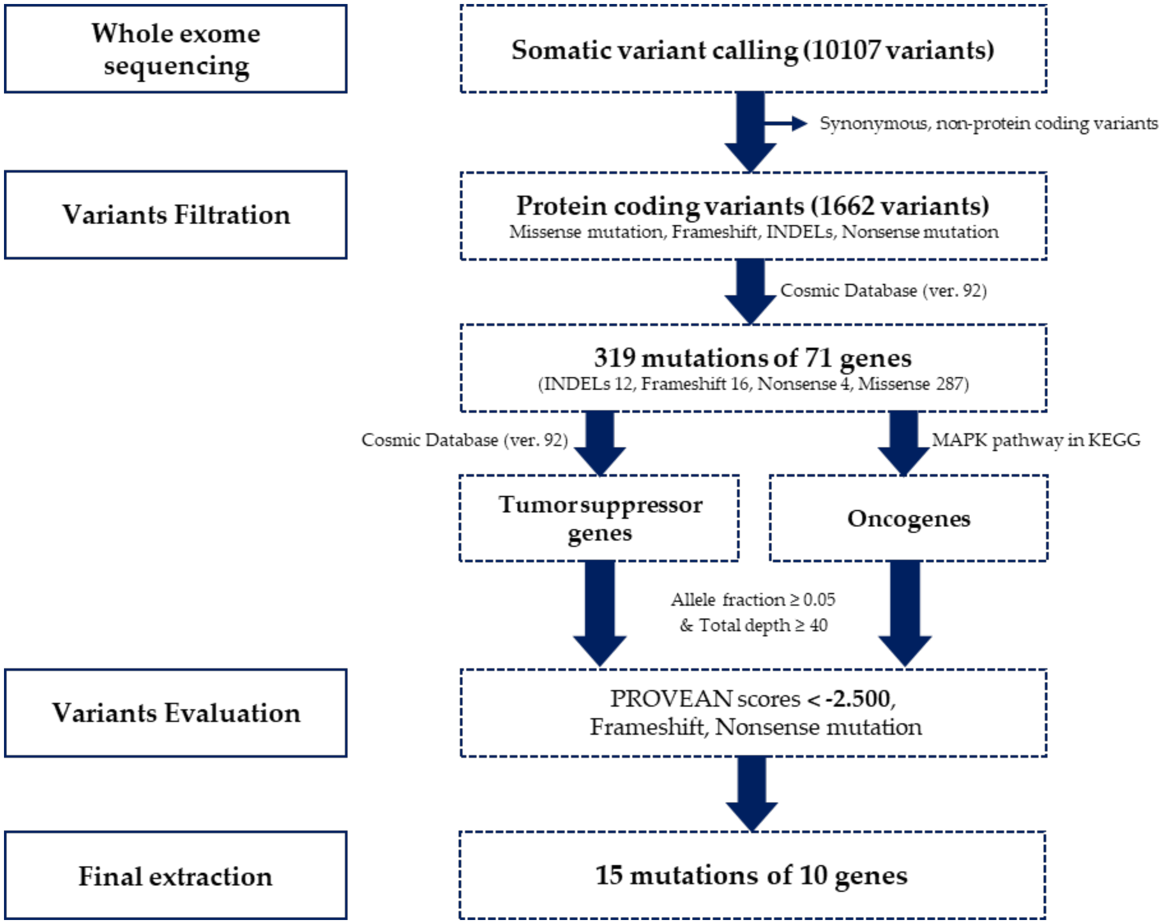

Supplementary Figure 2. Overall workflow of extracting significant somatic variants from whole exome sequencing.

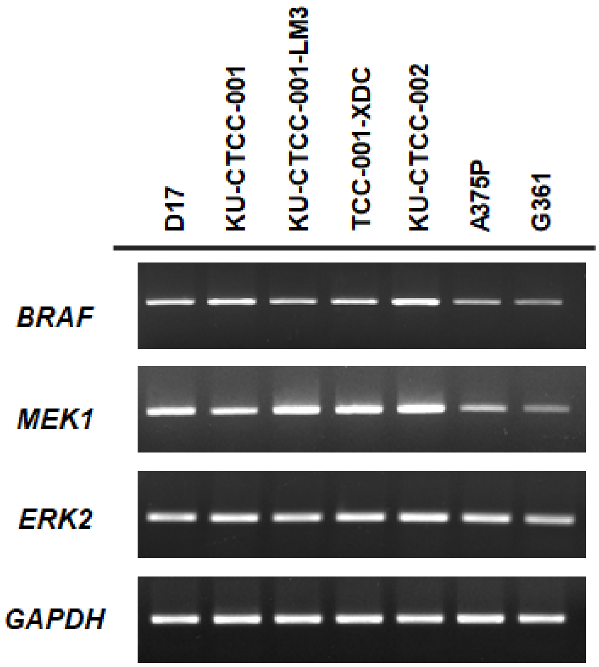

Supplementary Figure 3. Endogenous expression of BRAF/MAPK pathway genes. The mRNA expression levels of MEK1 and ERK2 were examined by RT-PCR.

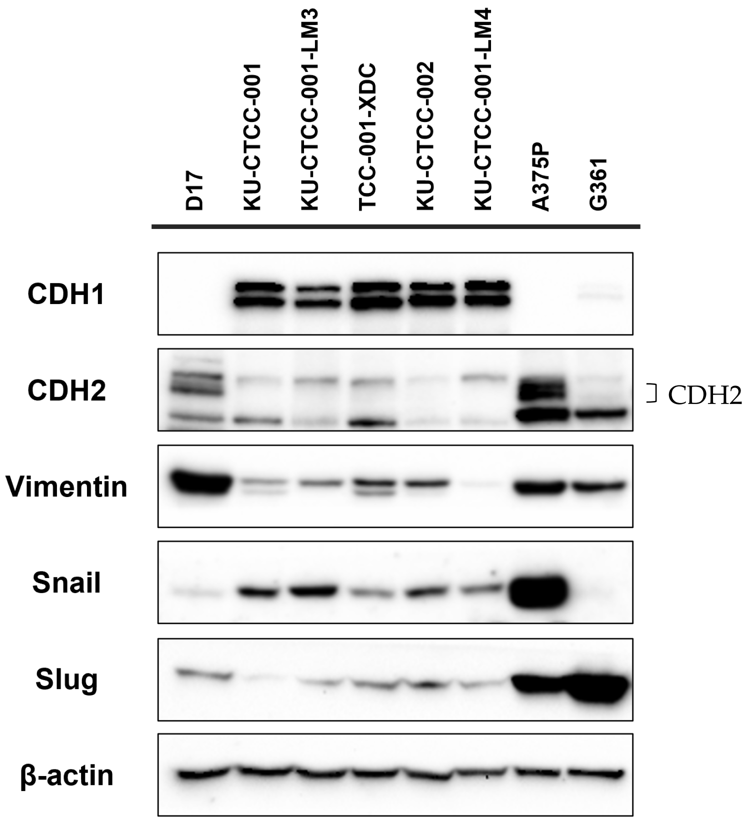

Supplementary Figure 4. Protein expression of EMT Markers. High expression of CDH1 and low expression of mesenchymal markers such as CDH2 and vimentin in canine TCC cell lines were detected by Western blot analysis.

A

B

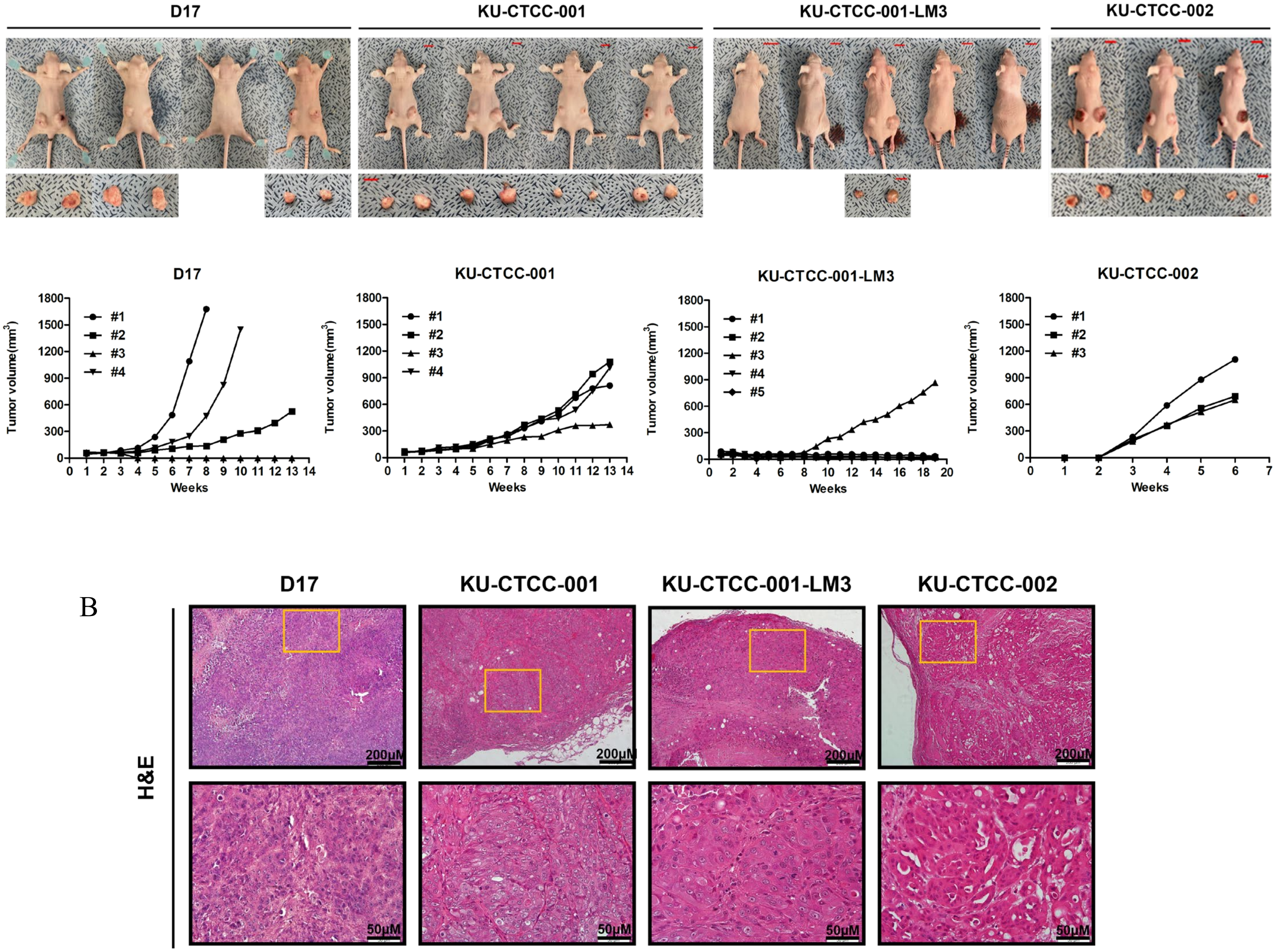

Supplementary Figure 5. Tumorigenicity in athymic nude xenografts. (A) Athymic nude xenograft mice and growth curve of the subcutaneous tumors. KU-CTCC-001 and KU-CTCC-002 exhibited 100% tumor formation in the xenografts, whereas D17 and KU-CTCC-001-LM3 cells did not. KU-CTCC-001-LM3 showed tumorigenicity in only one of five mice. Tumors injected with KU-CTCC-002 grew the fastest. Scale bar: 1 cm. (B) H&E stained images of subcutaneous tumors. The histologic features were the same as those of the NOG xenograft tumors.

Supplementary Table 1. Significant somatic variants of KU-CTCC-001.

Chromosome	Gene	Codon change	Amino acid change	Effect	Allele fraction	PROVEAN	
						Score	Predictions
16	***BRAF***	c.1763T>A	p.Val588Glu	missense_variant	0.43	-4.786	Deleterious
9	***NOTCH1***	c.5217G>T	p.Leu1739Phe	missense_variant	0.143	-2.756	Deleterious
16	***KMT2C***	c.7170_7171insG	p.Arg2391fs	frameshift_variant	0.12		
		c.7168delA	p.Thr2390fs	frameshift_variant	0.118		
31	***RUNX1***	c.1253T>G	p.Val418Gly	missense_variant	0.106	-2.736	Deleterious
		c.1262A>C	p.Glu421Ala	missense_variant	0.055	-3.504	Deleterious
X	***KDM5C***	c.166T>G	p.Phe56Val	missense_variant	0.104	-5.998	Deleterious
5	***NCOR1***	c.568C>T	p.Arg190*	stop_gained	0.085		
		c.534G>C	p.Lys178Asn	missense_variant	0.053	-4.236	Deleterious
3	***SLC34A2***	c.1454A>T	p.Gln485Leu	missense_variant & splice_region_variant	0.085	-6.819	Deleterious
9	***NF1***	c.1388A>G	p.Asp463Gly	missense_variant	0.073	-2.607	Deleterious
		c.1406A>G	p.Tyr469Cys	missense_variant	0.072	-4.714	Deleterious
26	***POLE***	c.1082A>G	p.His361Arg	missense_variant	0.066	-7.368	Deleterious
X	***BCOR***	c.3750_3751delCC	p.Ala1250fs	frameshift_variant	0.065		
		c.3745_3746insTT	p.Thr1249fs	frameshift_variant	0.063		
